# Significant variability in limb symmetry across four different knee flexion strength tests after anterior cruciate ligament reconstruction

**DOI:** 10.1002/jeo2.70932

**Published:** 2026-10-03

**Authors:** Johan Högberg, Mathias Wernbom, Axel Sundberg, Emilia Hermansson, Ted Larsson, Filip Adell, Kristian Samuelsson, Eric Hamrin Senorski

**Affiliations:** ^1^ Sportrehab Sports Medicine Clinic Gothenburg Sweden; ^2^ Sahlgrenska Sports Medicine Center Gothenburg Sweden; ^3^ Unit of Physiotherapy, Department of Health and Rehabilitation, Institute of Neuroscience and Physiology, Sahlgrenska Academy University of Gothenburg Gothenburg Sweden; ^4^ Department of Orthopaedics, Institute of Clinical Sciences, Sahlgrenska Academy University of Gothenburg Gothenburg Sweden; ^5^ Department of Orthopaedics Sahlgrenska University Hospital Mölndal Sweden

**Keywords:** ACL, hamstrings, knee flexors, reconstruction, strength assessment

## Abstract

**Purpose:**

To investigate knee flexion strength symmetry across four different strength tests in patients who underwent anterior cruciate ligament (ACL) reconstruction (ACLR) using hamstring tendon (HT) autograft.

**Methods:**

Data were collected between June 2024 and August 2025. Patients who underwent ACLR with HT autograft and performed (a) seated isokinetic concentric knee flexion (Biodex), (b) Nordic hamstring exercise (NordBord), (c) prone isometric 90° knee flexion with dorsiflexion (ISO90 DF) and (d) plantar flexion (ISO90 PF) ≥ 4 months after ACLR were included. The primary analysis was the cross‐sectional pairwise comparison of limb symmetry index (LSI) using a paired‐samples *t* test. The secondary analysis compared the proportion of patients achieving an LSI ≥ 90% in each testing modality using McNemar's test. Comparisons with a control group consisting of patients with patellar tendon and quadriceps tendon autografts were performed using an independent‐samples *t* test and Fisher's exact test, respectively. Statistical significance was set at *p* < 0.05.

**Results:**

A total of 101 patients were included, including a control group (*n* = 33). Patients with HT autograft had a mean age of 25 ± 8 years, with the most common follow‐up time being 12 months. Knee flexion strength in the Biodex displayed the LSI closest to symmetry (98.4%). The mean difference was 43.9% ± 16.7% (95% confidence interval [CI] [39.9; 47.8%], *p* < 0.001, *d* = 2.62 95% CI [2.1; 3.1]) compared to the LSI of the prone ISO90 PF. Fifty‐seven (80%) patients passed the ≥90% cut‐off in LSI for the Biodex test, while only 2 (3%) patients passed the prone ISO90 PF. The HT autograft group demonstrated lower LSI with the NordBord, ISO90 DF and ISO90 PF (*Δ* = −12.2 to −37.4%, *p* = 0.001 to <0.001) compared with the control group.

**Conclusions:**

Knee flexion strength LSI varied substantially across the four test modalities in patients with HT autograft, highlighting the need to consider the hip, knee and ankle positions when evaluating knee flexion strength recovery after ACLR in patients with HT autograft.

**Level of Evidence:**

Level II, prospective comparative study.

## Graphical abstract

Knee flexion strength symmetry after anterior cruciate ligament reconstruction with hamstring tendon autograft is highly dependent on the test modality.

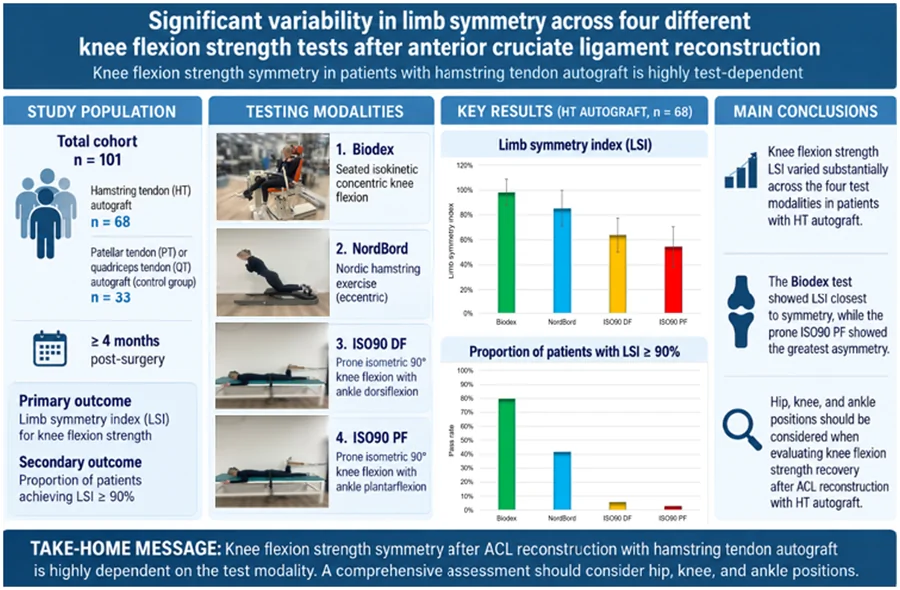

**Abbreviations**
ACL: anterior cruciate ligament
ACLR: anterior cruciate ligament reconstruction
BFlh: biceps femoris long head
CI: confidence interval
GR: gracilis
HT: hamstring tendon
IQR: interquartile range
ISO90 DF: prone isometric 90° knee flexion with ankle dorsiflexion test
ISO90 PF: prone isometric 90° knee flexion with ankle plantar flexion test
kg: kilogram
LSI: limb symmetry index
M: metres
*N*
: numbers
NHE: Nordic hamstring exercise
PCSA: physiological cross‐sectional area
PT: patellar tendon
QT: quadriceps tendon
SM: semimembranosus
ST: semitendinosus
Tegner: Tegner activity scale

## INTRODUCTION

Anterior cruciate ligament (ACL) injuries are common and devastating in physically active populations [5]. A hamstring tendon (HT) autograft, harvested from the semitendinosus (ST) tendon alone or together with the gracilis (GR), is widely used for ACL reconstruction (ACLR) due to favourable graft strength [29], and less anterior knee pain compared to patellar tendon (PT) autograft [30]. However, HT harvest results in knee flexion strength deficits [14] and alterations in knee flexor muscle morphology [7, 13].

Knee flexion strength recovery is important for rehabilitation progression, informing return‐to‐sport decisions and mitigating risk of re‐rupture [12, 19, 20]. Seated isokinetic concentric leg‐curl testing is the most commonly used assessment [33], but may not adequately reflect ST strength recovery. Increased compensatory activation of the biceps femoris long head (BFlh) and the semimembranosus (SM) may occur during dynamic knee flexion in a flexed hip position [31]. Thus, assessments of knee flexion strength only in a hip‐flexed position may fail to detect persistent knee flexion strength deficits [16, 17, 27], particularly in the ST muscle [31], which plays an important role in dynamic knee‐joint stabilization [23].

The Nordic hamstring exercise (NHE) assessed using a NordBord measures maximal eccentric knee flexion strength with the hip in extension and may unmask deficits not identified by seated isokinetic knee flexion strength assessments [16, 17]. However, as a maximal eccentric strength test, the NHE is technically challenging. The result is not only dependent on maximal eccentric strength, technique and coordination, but also on anthropometric factors such as body mass and body segment proportions (e.g., lower limb length relative to trunk length), which influence the external knee flexion moment during the NHE. Consequently, force output from the NHE may be less reliable in patients with lower knee flexion strength capacity and/or coordination ability.

Given the heterogeneous activation pattern of the knee flexors across exercises [4], strength tests that manipulate joint positioning, particularly by incorporating deeper knee flexion angles, hip extension and ankle plantar flexion, may more selectively target the ST muscle and reduce compensatory activation from the BFlh, SM [21] and the gastrocnemius [11]. A strength test protocol that manipulates these joint positions may provide a more sensitive assessment of eventual residual knee flexion strength deficits in patients after ACLR with HT autograft and inform more individualized rehabilitation strategies.

The aim of this study was to investigate knee flexion strength symmetry across four different strength tests in patients who were at least 4 months after their ACLR using HT autograft. The hypothesis was that knee flexion strength asymmetry would be greatest in a prone position at a deep knee flexion angle with the ankle positioned in plantarflexion.

## METHODS

### Study design

This prospective observational study followed the REporting of studies Conducted using Observational Routinely‐collected health Data (RECORD) guidelines [3] and was based on Project ACL, a rehabilitation‐focused registry established in 2014 including patient‐reported outcomes and standardized performance‐based tests. All patients provided written informed consent upon registration in the registry. Ethical approval was granted by the Swedish Ethical Review Authority (2020‐02501, 2024‐08724‐01) and the Regional Ethical Review Board in Gothenburg, Sweden (265‐13, T023‐17).

### Selection process

Patients in Project ACL were eligible if they had:

1. A confirmed ACL injury and subsequent ACLR using HT autograft.
2. Completed the registry's muscle function test battery at ≥4 months after ACLR.

Patients with contralateral HT harvest were excluded.

A control group was included with patients who had confirmed ACL injury and subsequent ACLR using either PT or quadriceps tendon (QT) autograft who participated in the registry's muscle function test battery after ACLR.

### Procedure

Between June 2024 and August 2025, eligible patients scheduled for follow‐up testing in the rehabilitation registry were contacted by phone prior to their test date to inform them about the study and to obtain consent to perform two additional isometric strength tests. For patients not reached beforehand, verbal consent was obtained during the testing session on site. Four different strength assessments, that is, isokinetic concentric knee flexion strength, eccentric knee flexion strength and two isometric knee flexion strength tests, were performed at a single testing session.

A complete‐case approach was applied for patients with HT autograft, only including patients who had complete data. Demographics and isokinetic strength testing were extracted from Project ACL. Three dichotomous questions were posed with regard to the presence of anterior knee pain, present symptoms in the hamstrings (without further clarification of symptoms) and previous strain/sudden sharp pain in the hamstring since ACLR. Results from the complementary isometric tests were recorded manually and compiled into a Microsoft Excel spreadsheet prior to statistical analysis.

### Knee flexion strength testing protocols

Isokinetic concentric knee flexion strength testing was conducted using the Biodex System 4 dynamometer (Biodex Medical Systems). The Biodex has an acceptable validity for peak torque assessment and high test‐retest reliability (intraclass correlation [ICC] = 0.82–0.99) [8, 10]. Testing was performed unilaterally at an angular velocity of 90°/s in the concentric mode, starting with the ACLR limb using a range of motion of 90°−0° of knee flexion. Patients were seated with the hip at 85° flexion, arms crossed over the chest and secured with straps across the thigh, pelvis and trunk to prevent compensatory movement. The mechanical arm was aligned with the knee joint axis, and the distal pad was applied just proximal to the lateral malleolus. Following a standardized warm‐up (10 min of cycling and submaximal practice trials), patients performed 3–4 single maximal repetitions, with 40 s rest between each repetition. The peak torque value in Newton metres (Nm) was used for analysis.

Eccentric knee flexion strength was assessed with the NHE using the NordBord system (Vald Performance). The NordBord has demonstrated excellent test–retest reliability for eccentric knee flexion strength assessments (ICC = 0.99) [28]. Patients were positioned in a kneeling position with the hip in extension. Each patient completed a warm‐up (two submaximal repetitions), followed by two sets of three maximal eccentric repetitions with 2 min of rest between sets. During each repetition, patients were instructed to control the descent as slowly as possible while keeping the hips extended and trunk neutral. Visual feedback on force production was provided between sets. Trials displaying compensatory hip motion or excessive lumbar extension were discarded. Force data were recorded using the NordBord software, and the maximum force in Newton (N) from an acceptable repetition was used for analysis.

After the Biodex and NordBord tests, two isometric strength tests were performed in a prone position with the knee fixed at 90° of flexion. Isometric knee flexion strength was assessed while the patient was verbally instructed to maintain the ankle in (1) maximal dorsiflexion and (2) plantar flexion, respectively. The patients were placed in a prone position on a treatment table with the pelvis stabilized using a belt to minimize hip flexion movement. The tested limb was secured with the knee flexed at 90°, and the contralateral limb rested in extension. A fixed dynamometer (Kern HCB 50K20; Kern & Sohn GmbH) with a strap just proximal to the lateral ankle was used. The Kern dynamometer has demonstrated excellent validity and intra‐ and interrater reliability for torque assessment (ICC = 0.88–0.96) [2]. Each patient performed three maximum voluntary isometric contractions for each ankle position, dorsiflexed and plantar flexed, on both the ACLR and uninjured limb, starting with the ACLR limb. For each condition, the patient was instructed to build up the maximum force over 5 s while maintaining the prescribed ankle position. Twenty seconds of rest were provided between each trial. Verbal encouragement was given to maximize effort. If any compensation or deviation from the target joint position occurred, the trial was discarded and repeated after rest.

The maximal force value (N) from the best trial in each condition (dorsiflexion and plantar flexion) was recorded for both limbs. The knee flexion strength tests applied are illustrated in Figure 1.

**Figure 1:**
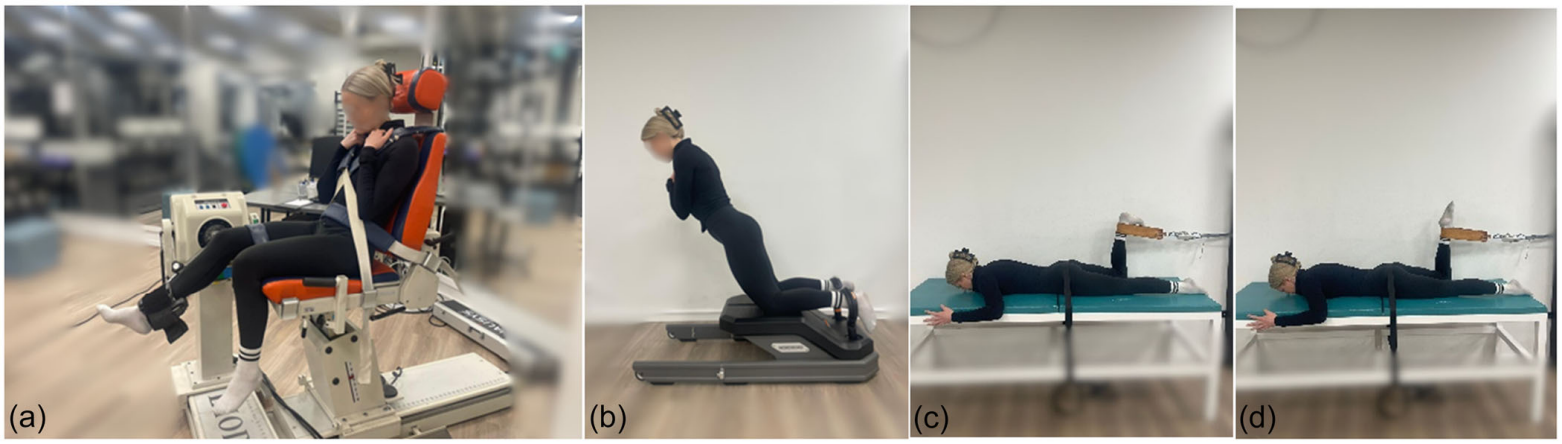
The knee flexion strength tests: (a) the Biodex, (b) the NordBord, (c) the ISO90 DF and (d) the ISO90 PF. ISO90 DF, prone‐lying isometric knee flexion test with the ankle in dorsiflexion; ISO90 PF, prone‐lying isometric knee flexion test with the ankle in plantar flexion.

### Outcome measures

Primary outcomes were the limb symmetry indices (LSIs) for knee flexion strength across the four testing modalities. The primary analysis was the pairwise comparison of LSI between (a) the seated isokinetic concentric knee flexion test (Biodex), (b) the NHE (NordBord), (c) the prone isometric 90° knee flexion test with dorsiflexion (ISO90 DF) and (d) with plantar flexion (ISO90 PF) in patients ≥4 months after ACLR using an HT autograft. The LSI was calculated by dividing the results of the ACLR limb by that of the uninjured limb, expressed as a percentage.

A secondary outcome was the proportion of patients achieving an LSI ≥ 90% in each testing modality. This cut‐off has been proposed as a threshold to be defined as a ‘successful outcome’ [20].

A third outcome comprised the absolute knee flexion strength values (Nm and N) obtained across the four testing modalities. These values were used to examine correlations between the absolute knee flexion strength values in the respective testing modality within each limb.

All outcomes were also investigated in a control group consisting of patients with PT and QT autografts. In addition, LSI values and the proportions achieving an LSI ≥ 90% for each testing modality were compared between the HT autograft and control groups.

### Statistical analysis

Data analysis was performed using IBM SPSS Statistics for Windows (Version 29.0; IBM Corp.). Descriptive statistics were reported as means ± standard deviations or median (interquartile range) with minimum and maximum values for continuous variables and as frequencies with proportions for categorical variables. In the demographics, patients with HT autografts and the control group were compared using independent‐samples *t* test for continuous variables and Fisher's exact test for categorical variables.

Normality of the respective variables and of the paired differences in LSI was assessed through visual inspection of histograms and using the Shapiro–Wilk test. Paired‐samples *t* tests were used to compare LSI values between the different knee flexion strength tests. Cohen's *d* was used to assess effect size, with thresholds of 0.2 = small, 0.5 = medium and 0.8 = large [6]. Comparisons in the proportion of patients reaching the ≥90% LSI threshold between testing methods were assessed pairwise using McNemar's test. Comparison of LSI between the HT autograft and control groups was performed using an independent‐samples *t* test and was presented with 95% confidence intervals (CIs). Comparison between groups in the proportion of patients achieving an LSI ≥ 90% was assessed using Fisher's exact test. Statistical significance was set at *p* < 0.05.

Data distribution was assessed visually with scatter plots to ascertain that no outliers would bias the Pearson's correlation coefficient analysis. Reference values for correlation coefficients were: 0.1–0.3 = weak, >0.3–<0.4 = weak‐to‐moderate, 0.4–0.6 = moderate, >0.6– <0.7 = moderate‐to‐strong, 0.7–0.9 = strong, >0.9–<1.0 = very strong and 1 = perfect [1]. Based on a clinically meaningful difference of 10% in LSI and an expected standard deviation of 15%, a sample size of 41 patients was determined to provide 80% power at a 0.05 significance level.

## RESULTS

A total of 101 patients were included, including the control group (*n *= 33). In the study cohort of patients with HT autografts, 68 patients (32% men) were included. The mean age at testing was 25 ± 8 years, and the median pre‐injury Tegner Activity Scale level was 9 (1–10). The median time of muscle strength testing after ACLR was 12 months. Three patients completed the assessments at two different follow‐ups, resulting in 71 unique muscle strength testing sessions. In the control group, one patient completed the assessment at two different follow‐ups, resulting in 34 muscle strength testing sessions. Due to discomfort/anterior knee pain during kneeling related to the donor‐site morbidity after tendon harvest, only 18 (55%) patients in the control group performed the NordBord assessment. No adverse events occurred during the strength assessments. Table 1 displays demographics of the included patients.

**Table 1:** Characteristics of patients included.

	HT autograft (*n* = 68)	Control group (*n* = 33)	*p* value
Men, *n* (%)	22 (32)	13 (39)	0.67
Age at time of testing, years	25 ± 8	26 ± 11	0.74
Height, m	1.71 ± 0.09	1.75 ± 0.08	0.04
Weight, kg	68 ± 11	74 ± 10	0.02
BMI, kg/m^2^	23 ± 3	24 ± 3	0.15
Tegner pre‐injury (IQR), median with minimum and maximum values	9 (7–9), 1–10	8 (8–9), 1–10	0.73
Graft type, n (%)	HT autograft: 68 (100)	PT autograft: 32 (97%); QT autograft: 1 (3%)	—
Time of muscle strength testing after ACLR in months, median (IQR) with minimum and maximum values	12 (10), 4–120	8 (10), 2–360	0.25
Anterior knee pain, *n* (%)	28 (39)	15 (44)	0.81
Symptoms in hamstrings, *n* (%)	28 (39)	1 (3)	<0.001
Previous sharp pain/strain in hamstrings, *n* (%)	16 (23)	6 (18)	0.84

*Note*: Mean presented with standard deviation unless otherwise stated.

Abbreviations: ACLR, anterior cruciate ligament reconstruction; BMI, body mass index; HT, hamstring tendon; IQR, interquartile range; kg, kilograms; m, metres; *n*, numbers; PT, patellar tendon; QT, quadriceps tendon; Tegner, Tegner activity scale.

### Comparison of knee flexion strength LSI

Knee flexion strength assessed with the Biodex displayed the most symmetrical LSI (98.4%). The mean difference was 13.1% ± 14.0% (95% CI [9.8; 16.4%], *p* < 0.001, *d* = 0.94 95% CI [0.7; 1.2]) compared with the NordBord LSI. The corresponding mean differences were 34.3% ± 15.2% (95% CI [30.7; 37.9%], *p* < 0.001, *d* = 2.26 95% CI [1.8; 2.7]) compared with the prone ISO90 DF LSI, and 43.9% ± 16.7% (95% CI [39.9; 47.8%], *p* < 0.001, *d* = 2.62 95% CI [2.1; 3.1]) compared with prone ISO90 PF LSI.

Knee flexion strength assessed with the NordBord displayed a mean LSI difference of 21.2% ± 13.8%, 95% CI [18.0; 24.5%], *p* < 0.001, *d* = 1.54, 95% CI [1.2; 1.9], compared with the prone ISO90 DF LSI. The mean difference was 30.7% ± 14.9%, 95% CI [27.2; 34.3%], *p* < 0.001, *d* = 2.01, 95% CI [1.7; 2.5], compared with the prone ISO90 PF.

Knee flexion strength in the prone ISO90 DF displayed a mean LSI difference of 9.5% ± 8.6%, 95% CI [7.5; 11.6%], *p* < 0.001, *d* = 1.11, 95% CI [0.8; 1.4], compared with the prone ISO90 PF (Figure 2).

**Figure 2:**
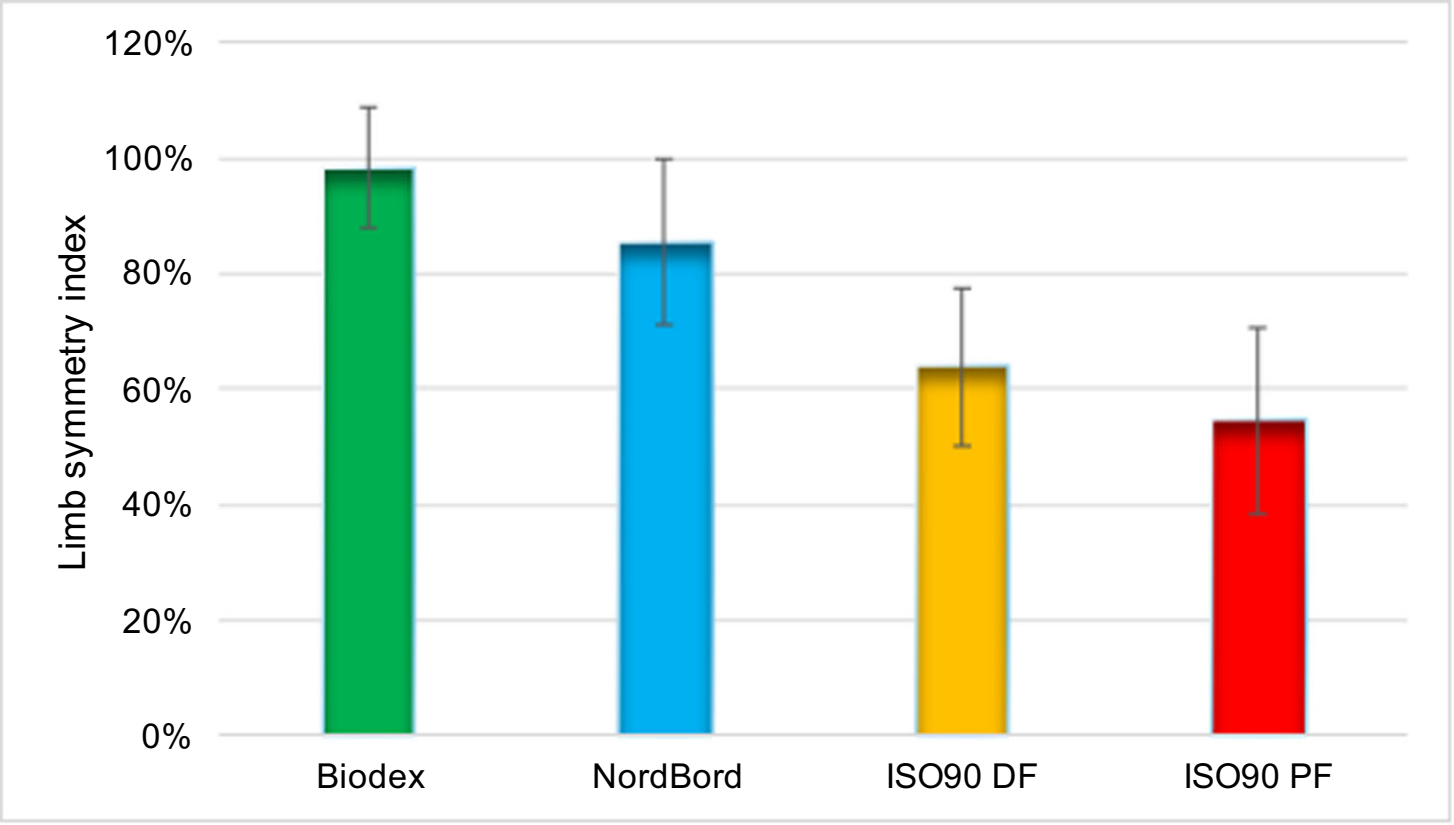
Knee flexion strength limb symmetry for each testing modality. All pairwise comparisons between the respective tests were *p* < 0.001. ISO90 DF, prone‐lying isometric knee flexion test with the ankle in dorsiflexion; ISO90 PF, prone‐lying isometric knee flexion test with the ankle in plantar flexion.

In the control group, there was a mean difference between the Biodex LSI and the prone ISO90 DF of 5.2% ± 14.4% (95% CI [0.1; 10.2%], *p* = 0.04, *d* = 0.36 95% CI [0.01; 0.70]), and with the prone ISO90 PF of 6.1% ± 12.8% (95% CI [1.7; 10.6%], *p* = 0.009, *d* = 0.48 95% CI [0.12; 0.83]). There was no other difference between the respective testing modalities (Supporting Information S1: Figure 1).

### Comparison of LSI ≥ 90% pass rates

Fifty‐seven (80%) patients passed the ≥90% cut‐off in LSI for the Biodex test, while only 2 (3%) patients passed the prone ISO90 PF. The Biodex had a greater pass rate than the NordBord (*Δ* = 43%, *p* < 0.001), the prone ISO90 DF (*Δ* = 75%, *p* < 0.001) and the prone ISO90 PF (*Δ* = 78%, *p* < 0.001). The NordBord had a greater passing rate than the prone ISO90 DF (*Δ* = 37%, *p* < 0.001) and the prone ISO90 PF (*Δ* = 40%, *p* < 0.001). There was no significant difference in the passing rate between the prone ISO90 DF and the prone ISO90 PF (*p* = 0.50) (Figure 3).

**Figure 3:**
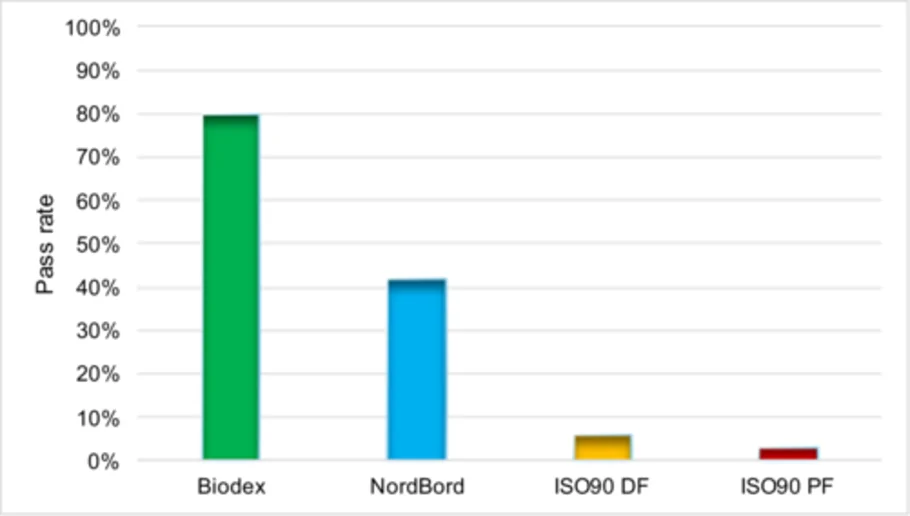
The proportion of patients who achieved ≥ 90% in limb symmetry index across each testing modality. The Biodex had a significantly greater passing rate than the NordBord and the prone isometric tests (*p* < 0.001). The NordBord had a significantly greater passing rate than the prone isometric tests (*p* < 0.001), while no significant difference in passing rate was observed between the prone isometric tests (*p* = 0.50). ISO90 DF, prone‐lying isometric knee flexion test with the ankle in dorsiflexion; ISO90 PF, prone‐lying isometric knee flexion test with the ankle in plantar flexion.

In the control group, 27 (79%) patients passed the ≥90% cut‐off in LSI for the Biodex test and 20 (59%) patients passed the prone ISO90 PF. The Biodex had a greater pass rate than the prone ISO90 DF (*Δ* = 32%, *p* = 0.007). No other differences in the passing rate were observed (Supporting Information S1: Figure 2).

### Correlations between knee flexion strength tests

Significant correlations were observed across all knee flexion strength tests, ranging from weak to very strong positive correlations. A weak‐to‐moderate positive correlation was observed between the absolute values of the uninjured limb measured with the NordBord and the prone ISO90 PF (*r* = 0.38, *p* = 0.001). A very strong positive correlation was found between the prone ISO90 DF and the prone ISO90 PF (*r* = 0.93, *p* < 0.001) for both the ACLR and uninjured limbs (Table 2).

**Table 2:** Correlations for both the ACLR and uninjured limb, across all testing modalities.

	Biodex	NordBord	ISO90 DF	ISO90 PF
ACLR limb				
Biodex		*r* = 0.66 *p* < 0.001	*r* = 0.55 *p* < 0.001	*r* = 0.48 *p* < 0.001
NordBord			*r* = 0.57 *p* < 0.001	*r* = 0.58 *p* < 0.001
ISO90 DF				*r* = 0.93 *p* < 0.001
ISO90 PF				
Uninjured limb				
Biodex		*r* = 0.69 *p* < 0.001	*r* = 0.56 *p* < 0.001	*r* = 0.48 *p* < 0.001
NordBord			*r* = 0.46 *p* < 0.001	*r* = 0.38 *p* = 0.001
ISO90 DF				*r* = 0.93 *p* < 0.001
ISO90 PF				

Abbreviations: ACLR, anterior cruciate ligament reconstruction; ISO90 DF, the isometric prone‐lying test with 90° of knee flexion and imposed dorsiflexion; ISO90 PF, the isometric prone‐lying test with 90° of knee flexion and imposed plantar flexion.

In the control group, weak to very strong positive correlations between the respective knee flexion strength tests were observed (Supporting Information S1: Table 1).

### Comparison between HT autograft and control groups

There was no difference in Biodex LSI between patients in the HT autograft and control groups (*Δ* = 0.4%, 95% CI [−4.1; 4.9%], *p* = 0.86). In contrast, patients with HT autograft demonstrated lower LSI with the NordBord, ISO90 DF and ISO90 PF (*Δ* = −12.2 to −37.4%, *p* = 0.001 to <0.001) compared with the control group (Table 3).

**Table 3:** Comparison of knee flexion strength LSI and ≥90% LSI pass rates between the HT autograft and control groups.

Test	HT autograft	Control group		
	LSI, mean ± SD	LSI, mean ± SD	Mean difference (95% CI)	*p* value
Biodex	98.4% ± 10.5%	98.0% ± 11.5%	0.4 (−4.1; 4.9)	0.86
NordBord	85.3% ± 14.4%	97.5% ± 10.3%	−12.2 (−19.4; −5.0)	0.001
ISO90 DF	64.1% ± 13.6%	92.8% ± 11.9%	−28.8 (−34.2; −23.3)	<0.001
ISO90 PF	54.5% ± 16.0%	91.9% ± 12.5%	−37.4 (−43.6; −31.2)	<0.001

	**LSI** ≥ **90%, *n* (%)**	**LSI** ≥ **90%, *n* (%)**	***p* value**	
Biodex	57 (80)	27 (79)	1.0	
NordBord	30 (42)	13 (72)	0.03	
ISO90 DF	4 (6)	16 (47)	<0.001	
ISO90 PF	2 (3)	20 (59)	<0.001	

*Note*: Analyses were based on 71 testing sessions from 68 patients with HT autograft and 34 testing sessions from 33 patients in the control group. NordBord data were available for 18 testing sessions in the control group.

Abbreviations: CI, confidence interval; HT, hamstring tendon; ISO90 DF, the isometric prone‐lying test with 90° of knee flexion and imposed dorsiflexion; ISO90 PF, the isometric prone‐lying test with 90° of knee flexion and imposed plantar flexion; LSI, Limb symmetry index; *n*, numbers; SD, standard deviation.

There was no difference between groups in the proportion of patients who achieved an LSI ≥ 90% with the Biodex (80% vs. 79%, *p* = 1.0). The proportion that achieved an LSI ≥ 90% was lower in patients with HT autograft for the NordBord (42% vs. 72%, *p* = 0.03), ISO90 DF (6% vs. 47%, *p* < 0.001), ISO90 PF (3% vs. 59%, *p* < 0.001) compared with the control group (Table 3).

## DISCUSSION

The main finding was significant variability in knee flexion strength LSI across the 4 different tests in patients after ACLR with HT autograft. The Biodex yielded the LSI closest to symmetry, whereas prone ISO90 PF had the greatest asymmetry, with a mean difference of ~44% LSI between these tests in the same patients. The comparison with the control group demonstrated no difference between groups in Biodex LSI, whereas progressively larger differences were observed with the NordBord, ISO90 DF and ISO90 PF, respectively. The largest between‐group difference was observed for the ISO90 PF, for which the HT autograft group demonstrated an LSI that was ~37 percentage points lower than the control group. In addition, there was a ~6% mean difference in LSI between the Biodex and ISO90 PF within the control group, suggesting that the significant variability in knee flexion strength LSI is specific to the HT autograft.

The varying degrees of correlation between tests suggest that, although all aim to assess knee flexion strength, they likely evaluate different aspects of knee flexion strength. These findings emphasize the importance of considering knee, hip and ankle joint angles when assessing knee flexion strength after ACLR in patients with HT autograft.

### Interpretation of key findings

A knee flexion strength LSI ≥ 90% has been reported as early as 4 months postoperatively [16, 32], despite ST tendon regeneration and maturation continuing up to 2 years after tendon harvest [7, 18]. A mean knee flexion strength LSI of ~98% in the Biodex, ~85% with the NordBord, ~64% with the prone ISO90 DF and ~55% with the prone ISO90 PF was observed at a median follow‐up of 12 months after ACLR. Variability in knee flexion strength LSI depending on the assessment methodology in patients treated with HT autograft for ACLR has previously been reported [27]. This variability across the four testing modalities is hypothesized to reflect the increased relative contribution of the ST muscle to the overall force production, with the prone ISO90 PF test likely isolating the ST the most. The ST has ~2–3 times longer muscle fibres than the BFlh and the SM, while the GR exhibits even longer fibres than the ST [22, 34, 35]. Consequently, the force‐generating capacity of the BFlh and SM during knee flexion is more influenced by hip angle compared with the ST and GR. Conversely, the SM and the BFlh muscles have greater physiological cross‐sectional areas (PCSA) than the ST and GR muscles [34, 35]. Accordingly, SM and BFlh are strong knee flexors at near‐optimum muscle lengths, corresponding to longer muscle lengths (e.g., hip flexion and close to knee extension) [34, 35]. In support of this view, knee flexion strength test with the hip in a flexed position has previously been reported to elicit relatively higher activation of the BFlh and reduced ST activity in the ACLR limb, potentially compensating for ST deficiency [31]. Furthermore, increased muscle volumes and PCSAs have been observed in the SM and BFlh muscles in the ACLR limb [18]. Collectively, differences in muscle and tendon architecture between the knee flexors appear to provide a potential explanation for how the SM and BFlh can largely compensate for defects in the ST and GR when knee flexion strength is assessed in a hip‐flexed position.

During the NHE, ST activation exceeds that of both the BFlh and the SM [4]. However, the BFlh and the SM may become increasingly active with decreased knee flexion, reaching approximately equal activation levels as the ST at angles below 50°–60° [15]. Thus, in patients who are able to resist through the entire range of motion in the NHE, the BFlh and the SM may, to some extent, mask underlying ST strength deficits, particularly when only maximum force is considered. Moreover, Nishida et al. [26] demonstrated that the ankle position in the NHE influences maximum force output, potentially due to varying contributions from the gastrocnemius muscle.

In addition to differences in joint position, the four tests differed in contraction mode. When considered according to contraction mode, the present results demonstrated the highest LSI during concentric testing (~98%), followed by eccentric testing (~85%), while the two isometric tests had the lowest LSI (~64% and ~55%). Contraction mode influences force‐generating capacity, with eccentric contractions generally allowing greater force production than isometric and concentric contractions due to the force‐velocity relationship [36]. Differences in absolute force‐generating capacity do not necessarily translate into differences in LSI, as the contralateral limb is used as the reference. Nevertheless, eccentric knee flexion strength LSI has previously been reported to be lower than concentric knee flexion strength LSI when both were assessed isokinetically [9], which suggests that contraction mode may influence the LSI. Contraction mode cannot be separated from joint position in the present study, as each contraction mode was assessed using different testing procedures and joint configurations. Nonetheless, the observed difference in LSI between the two isometric tests (~9%) despite an identical contraction mode in patients with HT autograft further indicates that contraction mode alone cannot explain the observed differences. Consequently, both contraction type and joint position should be considered when interpreting knee flexion strength symmetry in patients after ACLR with HT autograft.

### Clinical implications

Taking both contraction type and hip, knee and ankle joint positions into consideration when assessing knee flexion strength recovery in patients after ACLR with HT autograft may provide a more comprehensive evaluation of knee flexor function. In the present study, a high degree of symmetry during isokinetic concentric seated knee flexion strength testing did not necessarily indicate comparable recovery under eccentric or isometric testing conditions in other positions. Thus, relying on a single contraction type and position may leave residual deficits undetected. The observed variability in correlation between the different tests further emphasizes that no single test is sufficient to fully monitor knee flexion strength recovery. Given the bi‐articular nature and architectural differences of the knee flexor muscles, peak torque/maximum force is influenced not only by contractile capacity but also by joint position. When torque‐angle or force‐angle curves are available, strength deficits may be identified across the range of motion, potentially reducing the need for multiple isolated peak‐based tests. In settings where only peak values are accessible, a multifaceted testing approach is recommended to ensure a more accurate assessment of recovery. However, what LSI thresholds or absolute strength values to target during rehabilitation for the respective testing modalities remains uncertain and should be investigated in future research. Lastly, the clinical relevance of the persistent knee flexion strength deficit identified through the more targeted testing in the prone isometric tests remains uncertain.

### Methodological considerations

Given that ST regeneration and adaptation may continue for up to 2 years following surgery, LSI values, particularly for the prone tests, may vary over time. As the majority of patients were assessed at 8 and 12 months postoperatively, the external validity of our findings beyond 12 months after ACLR may be limited. Second, information on whether the ST tendon was harvested alone or together with the GR tendon was lacking. However, emerging data indicate that knee flexion strength recovery do not differ between using ST tendon alone or in combination with the GR tendon [24, 25]. Nonetheless, graft configuration may represent a potential confounding factor in our analysis. Third, multiple strength tests were performed within the same session, and fatigue may have influenced performance, particularly in later tests. The order of the Biodex and NordBord was standardized within the rehabilitation registry, precluding randomization. Furthermore, the tests differed in contraction mode (isometric, concentric, eccentric) and joint position. Consequently, it cannot be determined whether the observed differences between tests are primarily attributable to contraction type, joint position or their interaction, despite theoretical explanations based on hamstring muscle architecture. The control group consisted of patients who had undergone ACLR with PT or QT autograft rather than healthy individuals. Although this provides a clinically relevant comparison group without HT harvest, differences between autografts cannot be excluded. The control group was also relatively small, particularly for the NordBord assessment, for which data were available for only 18 patients. Consequently, the between‐group estimates should be interpreted with caution due to the reduced precision associated with smaller samples. Finally, variations in knee flexion range of motion, which were not accounted for, may have resulted in some patients being tested closer to their outer range than others, potentially influencing force production.

## CONCLUSION

Knee flexion strength LSI varied substantially across the four test modalities, where the Biodex test resulted in the LSI closest to symmetry and the prone ISO90 PF test the greatest asymmetry. The large differences in both LSI values and pass rates suggest that the investigated knee flexion strength tests assess different aspects of knee flexor strength and function.

## AUTHOR CONTRIBUTIONS

Johan Högberg drafted the initial version of the manuscript. Emilia Hermansson, Ted Larsson and Filip Adell contributed substantially to the acquisition of the data. Johan Högberg performed the analysis of the data. Johan Högberg and Eric Hamrin Senorski are responsible for drafting the manuscript and revising it critically for important intellectual content. Mathias Wernbom, Axel Sundberg and Kristian Samuelsson made large contributions to the revision and design of the work. Johan Högberg, Mathias Wernbom and Eric Hamrin Senorski are responsible for the concept of design.

## FUNDING INFORMATION

The authors have no funding to report.

## CONFLICT OF INTEREST STATEMENT

Kristian Samuelsson is a board member of Getinge AB. The results of the study are presented clearly, honestly and without fabrication, falsification or inappropriate data manipulation. The remaining authors declare no conflicts of interest.

## ETHICS STATEMENT

All patients received written and oral information about the project and provided informed consent. The study was approved by the Regional Ethical Review Board in Gothenburg (265‐13, T023‐17) and the Swedish Ethical Review Authority (2020‐02501, 2024‐08724‐01). Participation is voluntary, and informed consent is obtained during registration in the rehabilitation registry.

## Supporting information

Supporting file 1.

## Data Availability

Data are available upon reasonable request.
