# Supplementary material for: Significant variability in limb symmetry across four different knee flexion strength tests after anterior cruciate ligament reconstruction
Source: J Exp Orthop. 2026 Oct 3;13(4):e70932. doi: 10.1002/jeo2.70932 (PMC13633671; doi:10.1002/jeo2.70932)
Supplement: Supplementary file 1 [file JEO2-13-e70932-s001.docx]

**Supplementary files**

Limb symmetry index

|  | Limb symmetry index (%) |
| --- | --- |
| Biodex | 98% |
| NordBord | 97.5% |
| ISO90 DF | 92.8% |
| ISO90 PF | 91.9% |

**SUPPLEMENTARY FIGURE 1*.*** *Knee flexion strength limb symmetry for each testing modality for patients with PT and QT autograft. Only 18 patients had data for the NordBord. There was a significant difference between the Biodex LSI and ISO90 DF (p = 0.04) and with ISO90 PF (p = 0.009).* *ISO90DF = Prone-lying isometric knee flexion test with the ankle in dorsiflexion; ISO90 PF = Prone-lying isometric knee flexion test with the ankle in plantar flexion.*

Pass rate

|  | Pass |
| --- | --- |
| Biodex | 79% |
| NordBord | 72% |
| ISO90 DF | 47% |
| ISO90 PF | 59% |

**SUPPLEMENTARY FIGURE 2*.*** *The proportion of patients who achieved ≥90% in limb symmetry index across each testing modality.* *The Biodex had a significantly greater passing rate than the prone ISO90 DF (p = 0.007). ISO90DF = Prone-lying isometric knee flexion test with the ankle in dorsiflexion; ISO90 PF = Prone-lying isometric knee flexion test with the ankle in plantar flexion.*

**SUPPLEMENTARY TABLE 1*.*** Correlations for both the ACLR and uninjured limb in the control group, across all testing modalities.

| **ACLR limb** | | | | |
| --- | --- | --- | --- | --- |
|  | Biodex | NordBord | ISO90 DF | ISO90 PF |
| Biodex |  | *r* = 0.74  *p* < 0.001 | *r* = 0.54  *p* = 0.001 | *r* = 0.51  *p* = 0.002 |
| NordBord |  |  | *r* = 0.54  *p* = 0.02 | *r* = 0.45  *p* = 0.06 |
| ISO90 DF |  |  |  | *r* = 0.91  *p* < 0.001 |
| ISO90 PF |  |  |  |  |
| **Uninjured limb** | | | | |
|  | Biodex | NordBord | ISO90 DF | ISO90 PF |
| Biodex |  | *r* = 0.64  *p* = 0.005 | *r* = 0.72  *p* < 0.001 | *r* = 0.65  *p* < 0.001 |
| NordBord |  |  | *r* = 0.38  *p* = 0.12 | *r* = 0.29  *p* = 0.25 |
| ISO90 DF |  |  |  | *r* = 0.93  *p* < 0.001 |
| ISO90 PF |  |  |  |  |

*ACLR = Anterior cruciate ligament reconstruction ISO90 DF = the isometric prone-lying test with 90° of knee flexion and imposed dorsiflexion. ISO90 PF = the isometric prone-lying test with 90° of knee flexion and imposed plantar flexion.*
